# Association Between Opioid Dose Variability and Opioid Overdose Among Adults Prescribed Long-term Opioid Therapy

**DOI:** 10.1001/jamanetworkopen.2019.2613

**Published:** 2019-04-19

**Authors:** Jason M. Glanz, Ingrid A. Binswanger, Susan M. Shetterly, Komal J. Narwaney, Stan Xu

**Affiliations:** 1 Institute for Health Research, Kaiser Permanente Colorado, Aurora; 2 Department of Epidemiology, Colorado School of Public Health, Aurora; 3 Department of Biostatistics and Informatics, Colorado School of Public Health, Aurora; 4 Colorado Permanente Medical Group, Aurora; 5 Division of General Internal Medicine, Department of Medicine, University of Colorado School of Medicine, Aurora

## Abstract

**Importance:**

Attempts to discontinue opioid therapy to reduce the risk of overdose and adhere to prescribing guidelines may lead patients to be exposed to variability in opioid dosing. Such dose variability may increase the risk of opioid overdose even if therapy discontinuation is associated with a reduction in risk.

**Objective:**

To examine the association between opioid dose variability and opioid overdose.

**Design, Setting, and Participants:**

A nested case-control study was conducted in a large Colorado integrated health plan and delivery system from January 1, 2006, through June 30, 2018. Cohort members were individuals prescribed long-term opioid therapy.

**Exposures:**

Dose variability was defined as the SD of the milligrams of morphine equivalents across each patient’s follow-up and categorized based on the quintile distribution of the SD in the cohort (0-5.3, 5.4-9.1, 9.2-14.6, 14.7-27.2, and >27.2 mg of morphine equivalents).

**Main Outcomes and Measures:**

Opioid overdose cases were identified using *International Classification of Diseases, Ninth Revision* and *International Statistical Classification of Diseases and Related Health Problems, Tenth Revision *codes. Each case patient with overdose was matched to up to 20 control patients using risk set sampling. Conditional logistic regression models were used to generate matched odds ratios and 95% CIs, adjusted for age, sex, race/ethnicity, drug or alcohol use disorder, tobacco use, benzodiazepine dispensings, medical comorbidities, mental health disorder, opioid dose, and opioid formulation.

**Results:**

In a cohort of 14 898 patients (mean [SD] age, 56.3 [16.0] years; 8988 [60.3%] female) prescribed long-term opioid therapy, 228 case patients with incident opioid overdose were matched to 3547 control patients. The mean (SD) duration of opioid therapy was 36.7 (33.7) months in case patients and 33.0 (30.9) months in control patients. High-dose variability (SD >27.2 mg of morphine equivalents) was associated with a significantly increased risk of overdose compared with low-dose variability (matched odds ratio, 3.32; 95% CI, 1.63-6.77) independent of opioid dose.

**Conclusions and Relevance:**

Variability in opioid dose may be a risk factor for opioid overdose, suggesting that practitioners should seek to minimize dose variability when managing long-term opioid therapy.

## Key Points

**Question:**

Among individuals prescribed long-term opioid therapy, is variability in opioid dose associated with an increased risk of overdose?

**Findings:**

In this nested case-control study that included 228 case patients who experienced an opioid overdose and 3547 control patients who did not experience an opioid overdose, high variability in opioid dose was associated with a greater than 3-fold increased risk of opioid overdose even after controlling for dose.

**Meaning:**

The findings suggest that when managing long-term opioid therapy, practitioners should consider the risk of overdose associated with dose variability.

## Teaser

This nested case-control study of adults prescribed long-term opioid therapy examines the association between opioid dose variability and opioid overdose.

## Introduction

Epidemiologic studies^1,2,3,4,5^ have demonstrated that individuals prescribed high-dose opioid therapy are at increased risk for opioid overdose. These observational findings informed guidelines that encourage practitioners to minimize opioid prescribing for acute pain, to avoid initiating opioid therapy for chronic pain, and to consider tapering or discontinuing long-term opioid therapy when the risks of opioids outweigh the benefits.^6,7,8^ Although practice guidelines have led to substantial reductions in opioid prescribing across the United States,^6,7,8,9,10^ significant decreases in pharmaceutical opioid overdose have not been documented.^11^ It is thus possible that unexamined prescribing practices or unintended consequences of prescribing policies are contributing to persistently elevated pharmaceutical opioid overdose rates.^12^

Management of opioid therapy is a dynamic process that may result in a range of medication exposure patterns, including stable doses to changes in dose over time and therapy discontinuation. Changes in dose may include an increase, a decrease, or fluctuating increases and decreases over time. Studies of people initiating extended-release opioid treatment,^13^ initiating and discontinuing opioid agonist treatment for opioid use disorder,^14,15,16,17^ being released from prison,^18^ and being discharged from inpatient opioid use disorder treatment^19^ or hospitalization^20^ suggest that changes in dose may be associated with an increased risk of opioid overdose. The mechanisms that lead to these periods of risk are likely to be biological and behavioral, such as resuming opioid use after a period of reduced tolerance. To date, the association between dynamic changes in opioid dose or dose variability and opioid overdose has not been examined in the context of long-term opioid therapy.

The primary aim of this study was to examine the association between dose variability and the risk of opioid overdose. We also sought to quantify the association of sustained (≥3 months) opioid therapy discontinuation with opioid overdose. We hypothesized that increasing dose variability would be independently associated with opioid overdose and that dose variability would be more strongly associated with overdose at higher opioid doses. We also hypothesized that sustained opioid therapy discontinuation would be associated with a reduction in overdose risk.

## Methods

### Study Setting and Cohort

We conducted a matched case-control study that was nested within a cohort of Kaiser Permanente Colorado (KPCO) patients. KPCO is an integrated health care plan and delivery system with approximately 630 000 members. Cohort members were 18 years or older and were prescribed long-term opioid therapy, with 3 or more opioid dispensings of 10 mg or more of daily morphine equivalents in a 90-day period. Opioid dispensings were identified from KPCO pharmacy records and external pharmacy claims using National Drug Codes. Prescriptions had to be dispensed in the ambulatory setting from January 1, 2006, through December 31, 2017, with no more than 10 days without any opioid coverage. For each cohort member, milligrams of morphine equivalents were calculated for every month of follow-up using established methods.^4^ This study followed the Strengthening the Reporting of Observational Studies in Epidemiology (STROBE) reporting guideline. The KPCO Institutional Review Board approved the study before data collection, with a waiver of Health Insurance Portability and Accountability and informed consent. Data were not deidentified because this was a limited data set.

For entry into the long-term opioid cohort, buprenorphine-containing products were excluded because they are generally used for opioid use disorder treatment at KPCO. Methadone was included if it was dispensed at a pharmacy or if a pharmacy claim was generated; methadone dispensed in an addiction treatment center was excluded. Patients could have entered the cohort if they were already taking long-term opioid therapy in 2006 or if they newly started undergoing long-term opioid therapy from January 1, 2006, to December 31, 2017. The third opioid dispensing date of the 3 eligibility prescriptions represented the index dose. To be included in the cohort, patients needed at least 30 days of enrollment after their index dose. Patients were excluded from the cohort if they were in hospice, were in a nursing home, or had evidence of cancer during the follow-up. Patients were followed up until disenrollment from the health care plan, death, or June 30, 2018.

Across each patient’s follow-up, we assessed variability in dose by calculating the SD of the monthly milligrams of morphine equivalents over time from the index dose to the end of follow-up. A similar approach has been used to assess the association of heart rate variability and the risk of cardiovascular deaths.^21,22^ The dose variability exposure categories for the primary case-control analysis were based on the quintiles of the distribution of the SD in the cohort. Examining quintiles allowed us to examine a dose response between variability and overdose.

### Case and Control Patients

In the cohort, we identified cases of fatal and nonfatal opioid overdose using the *International Classification of Diseases*, *Ninth Revision* (*ICD-9*) and the *International Statistical Classification of Diseases and Related Health Problems, Tenth Revision (ICD-10)* codes (eTable 1 in the Supplement). These codes have an estimated positive predictive value of 81%^23^ and were identified in inpatient and emergency department settings or using state vital statistics. The date of the first overdose represented the index date.

Using risk set sampling,^24,25^ we randomly selected up to 20 control patients for each case patient who experienced overdose. This number was based on a sample size calculation that indicated the need for 100 case patients and 2000 matched control patients in the top and bottom quintiles and 2000 matched control patients to detect a matched odds ratio (mOR) of 2.05, with 80% statistical power, assuming an α of .05 and a probability of high variability in dose exposure of 0.2.

Eligible control patients did not have an *ICD-9* or *ICD-10* code for opioid overdose before the index date. Control patients were matched to case patients by milligrams of morphine equivalents at the index dose (±20%), calendar time (±60 days), and length of follow-up (±60 days). Length of follow-up was defined as the time between the index dose and index date. Each case patient and his or her set of matched control patients represented a separate stratum.

### Dose Variability, Sustained Opioid Therapy Discontinuation, and Dose for the Case-Control Analyses

For each case and control patient stratum, dose variability (SD), sustained opioid therapy discontinuation, and dose were assessed retrospectively from the index date. Dose variability was measured as the SD in dose between the index dose and the index date. For the primary analysis, the SD was categorized into the following groups based on the quintile distribution of SD in the cohort: 0 to 5.3, 5.4 to 9.1, 9.2 to 14.6, 14.7 to 27.2, and more than 27.2 mg of morphine equivalents.

Sustained therapy discontinuation was defined as 0 mg of morphine equivalents in the 3-month period before the index date and was coded as a dichotomous variable (yes or no). Dose was similarly calculated as the mean milligrams of morphine equivalents in the 3 months before the index date to capture the magnitude of dose in proximity to the overdose event. The doses were categorized as 0 to 20, 21 to 50, 51 to 100, and more than 100 mg of morphine equivalents.

### Statistical Analysis

#### Primary Analyses

Case patients and matched control patients were analyzed using conditional logistic regression to calculate mORs and 95% CIs. Two separate models were built: one for dose variability and one for sustained therapy discontinuation. In the first model, the dependent variable was overdose, and the main independent variable (exposure) was dose variability measured as a 5-level categorical variable (quintiles). The model was adjusted for the following variables, which have been reported in prior studies^2,4,5,13,26,27,28,29^ to be associated with overdose risk: opioid dose in the 3 months before the index date, age, sex, receipt of an extended-release and/or long-acting opioid formulation, mental health disorder diagnosis, drug or alcohol use disorder, benzodiazepine dispensings, medical comorbidity (Quan-Deyo Modified Charlson Comorbidity Index^30,31^), and tobacco use documented in the social history or tobacco use disorder as a diagnosis. Race/ethnicity was also included; data on race/ethnicity are populated in the KPCO electronic health record from patient self-report. Missing tobacco, race, and Hispanic ethnicity values were replaced using multiple imputations based on the available information from the matched sample. A dose-response association was tested for dose variability by modeling the variability categories (quintiles 1-5) as a continuous variable. To examine the opioid dose independent of dose variability, we analyzed dose without the dose variability measure in the model. To evaluate whether dose variability varied by the magnitude of the dose, dose variability and dose were also analyzed as an interaction. Model fit was assessed with the Hosmer-Lemeshow goodness-of-fit test on unmatched data,^32^ and multicollinearity was evaluated using the variance inflation factor.

The second multivariable conditional logistic regression model was built with overdose as the outcome and sustained therapy discontinuation as the main exposure variable, adjusting for the same covariates as the first model except for opioid dose in the 3 months before the index date.

#### Secondary Analyses

We conducted 4 secondary analyses on dose variability, which all controlled for the same variables as the primary variability analysis. First, dose variability (SD) was modeled as a continuous variable. Second, to evaluate the association of the most recent opioid dose change with overdose, we modeled the net change in milligrams of morphine equivalents in the month immediately preceding the index date. Third, to account for increasing and decreasing trends in dose, we fit a linear regression of opioid dose for the case patients during their follow-up time. On the basis of the dose trend, we created 2 groups: decreasing or flat (coefficient for the linear trend ≤0) and increasing (coefficient for linear trend >0). We then modeled the dose trend as a dichotomous variable (decreasing or flat vs increasing). Fourth, to assess the timing of dose variability relative to the index date, we conducted a separate regression analysis that evaluated the association between dose variability in 3-, 6-, 9-, and 12-month periods before the index date and overdose. Dose variability was modeled as a continuous variable for these latter analyses.

We also conducted a secondary analysis on therapy discontinuation that controlled for the same variables as the primary therapy discontinuation analysis. In this secondary analysis, we examined the association between therapy discontinuation and overdose when use of opioids was discontinued only 1 month before the index date.

#### Sensitivity Analyses for Differential and Nondifferential Outcome Misclassification

A multiple-imputation method was used to address the uncertainty introduced by potential outcome (case) misclassification.^33,34,35^ On the basis of the literature, we assumed an overall 19% misclassification rate for the *ICD-9* or *ICD-10* overdose diagnoses. We used Monte Carlo simulation (5000 replications) to determine the levels of outcome misclassification that would have affected our conclusion on the association between dose variability and overdose. We simulated both nondifferential and differential outcome misclassification by dose variability (exposure) status.

All analyses were conducted using SAS statistical software, version 9.4 (SAS Institute Inc). All statistical tests were 2-sided, and *P* < .05 was considered statistically significant.

## Results

A cohort of 14 898 patients (mean [SD] age, 56.3 [16.0] years; 8988 [60.3%] female) was prescribed long-term opioid therapy (Table 1). The Figure describes the cohort inclusions and exclusions. During the follow-up, 10 885 individuals (73.1%) had a mental health disorder diagnosis, 4816 (32.3%) had a drug or alcohol use disorder, 5089 (34.2%) used tobacco or had a tobacco use disorder, and 12 887 (86.5%) had a chronic pain diagnosis. The index dose was greater than 50 mg of morphine equivalents for 4064 individuals (27.3%) in the cohort.

**Table 1.** Demographic and Clinical Characteristics of the Total Cohort, Case Patients Who Experienced Overdose, and Control Patients^a^

Characteristic	Total Cohort (N = 14 898)	Case Patients (n = 228)	Control Patients (n = 3547)
Age, y			
≤45	3645 (24.5)	63 (27.6)	735 (20.7)
46-55	3471 (23.3)	55 (24.1)	977 (27.5)
56-65	3419 (22.9)	60 (26.3)	854 (24.1)
>65	4363 (29.3)	50 (21.9)	981 (27.7)
Follow-up, mean (SD), mo	54.5 (41.2)	39.7 (34.7)	37.4 (33.3)
Female	8988 (60.3)	145 (63.6)	2186 (61.6)
Race			
White	10 785 (72.4)	195 (85.5)	3119 (87.9)
African American	746 (5.0)	14 (6.1)	149 (4.2)
Other	1099 (7.4)	19 (8.3)	279 (7.9)
Missing	2268 (15.2)	NA^b^	NA^b^
Hispanic ethnicity	2167 (14.6)	29 (12.7)	399 (11.3)
Insurance			
Commercial	7217 (48.4)	103 (45.2)	1838 (51.8)
Medicare	5149 (34.6)	92 (40.4)	1293 (36.5)
Medicaid	1796 (12.1)	24 (10.5)	287 (8.1)
Other	736 (4.9)	9 (3.9)	129 (3.6)
Mental health disorder diagnosis	10 885 (73.1)	217 (95.2)	2820 (79.5)
Opioid medication formulation			
Short acting	12 054 (80.9)	126 (55.3)	2381 (67.1)
Long acting, extended release, or both	2844 (19.1)	102 (44.7)	1166 (32.9)
Drug or alcohol use disorder	4816 (32.3)	159 (69.7)	1508 (42.5)
Tobacco use or use disorder			
Former	3929 (31.5)	56 (24.6)	1001 (28.2)
Yes	5089 (34.2)	109 (47.8)	1179 (33.2)
No	5747 (38.6)	63 (27.6)	1367 (38.5)
Missing	133 (0.9)	NA^b^	NA^b^
Benzodiazepine dispensing	5468 (36.7)	105 (46.1)	961 (27.1)
Modified Charlson Comorbidity Index, mean (SD)	1.3 (1.8)	2.2 (2.3)	1.4 (1.8)
Morphine equivalent, mg^c^			
0-20	4965 (33.3)	39 (17.1)	1086 (30.6)
21-50	6039 (40.5)	54 (23.7)	1043 (29.4)
51-100	2443 (16.4)	56 (24.6)	679 (19.1)
>100	1451 (9.7)	79 (34.7)	739 (20.8)
Variability in dose			
0-5.3	2977 (20)	15 (6.6)	511 (14.4)
5.4-9.1	2977 (20)	20 (8.8)	540 (15.2)
9.2-14.6	2978 (20)	27 (11.8)	649 (18.3)
14.7-27.2	2977 (20)	52 (22.8)	805 (22.7)
>27.2	2977 (20)	114 (50.0)	1042 (29.4)
3-mo Therapy discontinuation^d^	NA	12 (5.3)	356 (10.0)
Overdose events	305 (2.0)	228 (100)	0

Abbreviation: NA, not available.

^a^ Data are presented as number (percentage) of study participants unless otherwise indicated.

^b^ Missing values were imputed in the regression models using multiple imputation.

^c^ Defined across first 3 prescriptions after the index dose for cohort and in 3 months before the index date for case and control patients.

^d^ Defined as 3 months before the index date without an opioid dispensing (0 mg per morphine equivalent) for case-control analysis.

**Figure.**
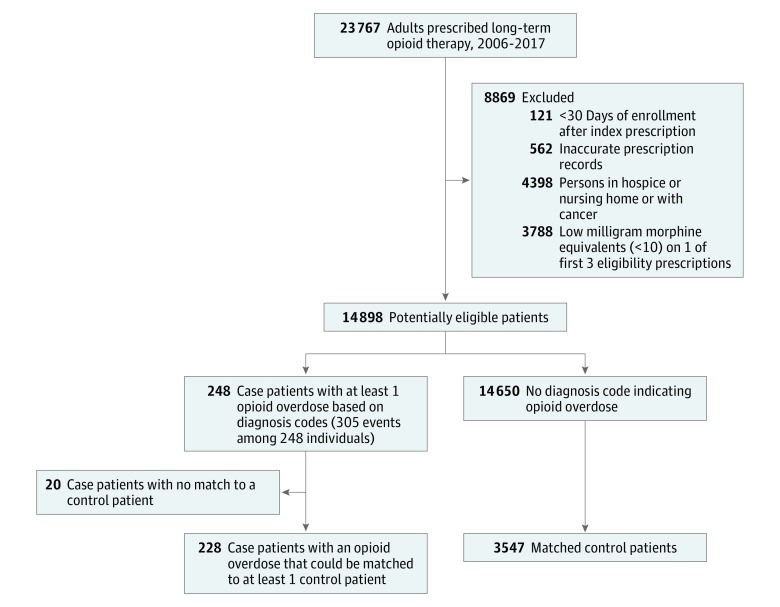
Cohort Flow and Nested Case-Control Study

In the cohort of patients prescribed long-term opioid therapy, we identified 305 opioid overdose events (incidence rate, 456 per 100 000 person-years). Of these events, 57 were recurrent (in 42 individuals), 26 were fatal, and 39 occurred during a suicide attempt. Of 248 case patients with incident overdoses, 228 could be matched to at least 1 control. Of 14 650 potential control patients, case patients were matched to 3547 control patients. Table 1 gives the characteristics of the case and control patients. Four patients had overdoses that involved heroin, and 224 had overdoses primarily attributable to pharmaceutical opioids. The mean (SD) time undergoing opioid therapy before the index date was 36.7 (33.7) months among case patients and 33.0 (30.9) months among control patients, and the mean (SD) opioid doses before the index date were 110.6 (133.8) mg of morphine equivalents for case patients and 73.0 (112.9) mg of morphine equivalents for control patients.

In the first multivariable conditional logistic regression analysis, dose variability and dose were independently associated with overdose (Table 2). Individuals exposed to the highest category of dose variability (SD >27.2 mg of morphine equivalents) had an mOR of 3.32 (95% CI, 1.63-6.77) for experiencing an overdose compared with individuals exposed to the lowest category of dose variability (SD ≤5.3 mg of morphine equivalents). Individuals prescribed high doses (>100 mg of morphine equivalents) in the 3 months before the index date had an mOR of 2.37 (95% CI, 1.41-3.98) for experiencing an overdose compared with individuals prescribed lower doses (0-20 mg of morphine equivalents). The analysis evaluating variability in dose as dose response was statistically significant (β = 0.30; 95% CI, 0.14-0.45). Without dose variability in the model, an opioid dose greater than 100 mg of morphine equivalents was associated with a higher risk of overdose (mOR, 3.10; 95% CI, 1.85-5.19) compared with a dose of 0 to 20 mg of morphine equivalents. An interaction was not found between dose and variability in dose (χ^2^_12_ = 8.81; *P* = .72), suggesting that the association of dose variability with overdose risk did not vary by the magnitude of dose. The Hosmer-Lemeshow test on the unmatched data indicated that model fit was adequate (χ^2^_8_ = 5.49; *P* = .70). The tolerance was 0.74, and the variance inflation factor was 1.35, indicating an absence of multicollinearity between dose variability and dose.^36^

**Table 2.** Unadjusted and Adjusted Associations Between Opioid Dose Variability and Opioid Overdose

Characteristic	mOR (95% CIs)^a^	
	Unadjusted	Adjusted
Variability in dose, SD		
0-5.3	1 [Reference]	1 [Reference]
5.4-9.1	1.62 (0.79-3.35)	1.43 (0.68-3.01)
9.2-14.6	2.02 (1.00-4.09)	1.61 (0.79-3.31)
14.7-27.2	3.54 (1.79-6.98)	2.19 (1.08-4.43)
>27.2	6.89 (3.48-13.61)	3.32 (1.63-6.77)
Dose, mg of morphine equivalents^b^		
0-20	1 [Reference]	1 [Reference]
21-50	1.68 (1.08-2.61)	1.53 (0.97-2.40)
51-100	3.36 (2.08-5.42)	2.27 (1.39-3.70)
>100	4.67 (2.83-7.72)	2.37 (1.41-3.98)
Age, y		
≤45	1 [Reference]	1 [Reference]
46-55	0.66 (0.45-0.97)	0.64 (0.42-0.96)
56-65	0.82 (0.56-1.20)	0.83 (0.55-1.25)
>65	0.65 (0.44-0.96)	0.73 (0.45-1.18)
Sex		
Male	1 [Reference]	1 [Reference]
Female	0.90 (0.68-1.19)	0.87 (0.64-1.18)
Hispanic ethnicity		
Yes	1 [Reference]	1 [Reference]
No	0.88 (0.58-1.34)	0.88 (0.55-1.41)
Race		
White	1 [Reference]	1 [Reference]
Black	1.55 (0.88-2.72)	1.43 (0.77-2.65)
Other	1.20 (0.74-1.94)	1.37 (0.80-2.36)
Drug or alcohol use disorder		
No	1 [Reference]	1 [Reference]
Yes	3.09 (2.29-4.17)	2.18 (1.58-3.00)
Tobacco use or use disorder		
No	1 [Reference]	1 [Reference]
Yes	1.99 (1.44-2.75)	1.68 (1.19-2.37)
Former	1.21 (0.83-1.75)	1.05 (0.71-1.56)
Mental health disorder diagnosis		
No	1 [Reference]	1 [Reference]
Yes	4.96 (2.68-9.18)	2.97 (1.57-5.64)
Opioid formulation		
Short acting	1 [Reference]	1 [Reference]
Long acting, extended release, or both	1.57 (1.09-2.28)	1.23 (0.83-1.84)
Benzodiazepine dispensing		
No	1 [Reference]	1 [Reference]
Yes	2.48 (1.86-3.30)	1.90 (1.40-2.59)
Modified Charlson Comorbidity Index	1.21 (1.14-1.28)	1.25 (1.16-1.35)

Abbreviation: mOR, matched odds ratio.

^a^ Matched on index dose, calendar time, and length of follow-up.

^b^ In the 3 months before the index date (date of overdose from case patients and matched date for control patients).

Individuals with sustained opioid therapy discontinuation (defined as 3 continuous months with 0 mg of morphine equivalents before the index date) were 51% less likely to have experienced an overdose than individuals who had not discontinued opioid therapy (mOR, 0.49; 95% CI, 0.26-0.93) (Table 3).

**Table 3.** Unadjusted and Adjusted Associations Between Sustained (3-Month) Opioid Therapy Discontinuation and Opioid Overdose

Characteristic	mOR (95% CIs)^a^	
	Unadjusted	Adjusted
Sustained opioid therapy discontinuation^b^		
No	1 [Reference]	1 [Reference]
Yes	0.42 (0.23-0.77)	0.49 (0.26-0.93)
Age, y		
≤45	1 [Reference]	1 [Reference]
46-55	0.66 (0.45-0.97)	0.63 (0.42-0.95)
56-65	0.82 (0.56-1.20)	0.86 (0.56-1.30)
>65	0.65 (0.44-0.96)	0.70 (0.44-1.13)
Sex		
Male	0.90 (0.68-1.19)	0.89 (0.65-1.21)
Female	1 [Reference]	1 [Reference]
Hispanic ethnicity		
Yes	1 [Reference]	1 [Reference]
No	0.88 (0.58-1.34)	0.96 (0.60-1.54)
Race		
White	1 [Reference]	1 [Reference]
Black	1.55 (0.88-2.72)	1.28 (0.69-2.41)
Other	1.20 (0.74-1.94)	1.34 (0.78-2.31)
Drug or alcohol use disorder		
No	1 [Reference]	1 [Reference]
Yes	3.09 (2.29-4.17)	2.38 (1.73-3.27)
Tobacco use or use disorder		
No	1 [Reference]	1 [Reference]
Yes	1.99 (1.44-2.75)	1.67 (1.19-2.36)
Former	1.21 (0.83-1.75)	1.07 (0.72-1.58)
Mental health disorder diagnosis		
No	1 [Reference]	1 [Reference]
Yes	4.96 (2.68-9.18)	2.72 (1.43-5.18)
Opioid formulation		
Short acting	1 [Reference]	1 [Reference]
Long acting, extended release, or both	1.57 (1.09-2.28)	1.42 (0.95-2.12)
Benzodiazepine dispensing		
No	1 [Reference]	1 [Reference]
Yes	2.48 (1.86-3.30)	3.63 (2.61-5.06)
Modified Charlson Comorbidity Index	1.21 (1.14-1.28)	1.26 (1.17-1.36)

Abbreviation: mOR, matched odds ratio.

^a^ Matched on index dose, calendar time, and length of follow-up.

^b^ In the 3 months before the index date (date of overdose from case patients and matched date for control patients).

In the secondary analyses, dose variability measured as a continuous variable was associated with overdose (mOR, 1.01; 95% CI, 1.00-1.01; *P* = .02) (eTable 2 in the Supplement). The change in opioid dose 1 month before the index date was not associated with overdose (mOR, 1.00; 95% CI, 1.00-1.00; *P* = .36). An increasing dose trend was not associated with overdose compared with a flat or decreasing dose trend (mOR, 1.11; 95% CI, 0.77-1.58). In the analyses examining dose variability in the 3-, 6-, 9-, and 12-month periods before the index date, dose variability measured as a continuous variable was associated with overdose (mOR, 1.01; 95% CI, 1.00-1.01 for each; 3 months, *P* = .003; 6 months, *P* = .001; 9 months, *P* = .002; 12 months, *P* = .005). The estimate for therapy discontinuation 1 month before the index date was similar to the estimate for discontinuation 3 months before the index date (mOR, 0.51; 95% CI, 0.30-0.87).

The sensitivity analyses assuming 19% nondifferential misclassification of outcome status (overdose classification) changed the main effect estimates by 2.7% or less and did not change the main conclusion that high-dose variability (SD, >27.2) was positively associated with overdose (eTable 3 in the Supplement). When assuming 19% differential misclassification of outcome status by dose variability status, the estimate for dose variability was reduced by approximately 30% under the most extreme differential misclassification scenarios. The main conclusion did not change under any of the misclassification scenarios.

## Discussion

In this case-control study of patients prescribed long-term opioid therapy, there was an apparent dose-response association between variability in opioid dose and overdose risk. Opioid dose also demonstrated an association with short-term overdose risk, whereas sustained opioid therapy discontinuation was associated with an approximate 50% reduction in risk of overdose. Together, these findings suggest that tapering patients off long-term opioid therapy may be beneficial if it ultimately leads to discontinuation of opioid therapy, but they also suggest that attempts to modify opioid doses could lead to dose variability that may be associated with increased risk of opioid overdose.

Although we hypothesized that dose variability would be associated with higher risk in patients receiving high opioid doses, the interaction between dose variability and dose was not statistically significant. This finding suggests that even among patients receiving low doses of opioids, dose variability is a risk factor for overdose. The results of our secondary analyses suggest that our primary results were robust to measuring variability as a continuous variable, the timing of the variability, the overall dose trend, and the timing of the discontinuation. Future research could focus on how to use measures of dose variability to guide prescribing practices.

Although there could be numerous reasons why patients are exposed to dose variability, one reason is an attempt to taper or discontinue opioid therapy. An attempted dose reduction may be associated with opioid withdrawal or increased short-term pain,^37,38,39^ prompting patients to request a subsequent dose increase. Because of loss of tolerance after a period of reduced opioid exposure,^40,41,42^ such dose adjustments may be associated with increased risk of opioid overdose. Alternatively, after a dose reduction, patients may seek pharmaceutical opioids from other sources (eg, friends) without being aware of the potency of the opioid formulation they are ingesting, increasing the risk of overdose. Future research should specifically examine the effects of tapering on overdose.

Although our results corroborate prior epidemiologic studies^2,4,5,26,36^ demonstrating that opioid doses above 50 mg of morphine equivalents are associated with estimated 2- to 6-fold increased short-term risks of overdose among patients prescribed long-term opioid therapy, the dose association was attenuated (30.8%) when dose variability was included in the analyses. This finding suggests that prior analyses assessing the association between opioid dose and overdose may have been confounded by dose variability. Prior research^27^ has also found that opioid dose is not associated with the long-term (2-year) risk of overdose. Given that dose is likely to fluctuate over time, the poor predictive value of dose for 2-year overdose risk may be partially explained by dose variability.

### Strengths and Limitations

This population-based study has several strengths. We assembled a cohort of longitudinally followed up patients receiving long-term opioid therapy in a large integrated health plan that is demographically representative of Colorado. In the cohort, overdose events were identified in the inpatient and emergency department settings and in vital records, and the estimated incidence rate of overdose was similar to or higher than rates among patients prescribed long-term opioids in prior studies.^4,43^ With use of a risk set sampling approach, randomly selected control patients were matched to case patients who experienced overdose by calendar time and length of follow-up time to help control for time-varying factors, such as secular trends in opioid prescribing policies and practices, potency and availability of illicit opioids, and health care system–specific overdose reduction interventions implemented across the follow-up period. In addition, the conditional logistic regression models were adjusted for several important potential confounding variables that are known risk factors for overdose.

This study also has limitations. Although the *ICD-9* and *ICD-10* overdose codes are associated with a high positive predictive value, up to 19% of the overdose cases may have been misclassified as false-positive. This misclassification could have been nondifferential or differential with respect to exposure status, leading to biased OR estimates. However, in our sensitivity analyses, we found that a wide range of exposure misclassification would not have changed our conclusion that dose variability may be associated with an increased risk of overdose among patients prescribed long-term opioid therapy.

Another potential limitation is loss to follow-up. It is possible that the patients who experienced dose variability may have sought illicit opioids such as heroin, lost their insurance, and experienced an overdose during a period of disenrollment from the health care plan. In our data, this would have appeared to be an exposed noncase when in fact it was an exposed case, implying that our observed OR was an underestimate of the true association. There could be multiple reasons why people experienced dose variability, including worsened pain, practitioner-initiated tapers, changing physicians, missed appointments, poor adherence to urine toxicology screening, or travel. If one of these reasons was driving the association, the observed association may have been diluted by combining them. Future research should examine specific reasons for dose variability and other potential outcomes, such as suicide.^44^

## Conclusions

This study suggests that dose variability is a risk factor for opioid overdose independent of dose alone, whereas sustained therapy discontinuation may be protective of overdose. Additional studies are needed to better understand the pathways by which patients undergoing long-term opioid therapy can safely discontinue opioid therapy. Until such pathways can be elucidated, policymakers and physicians should consider the risks that may be associated with dose variability when designing and implementing new policies to reduce opioid prescribing.

## Notes

Supplement. **eTable 1.** *International Classification of Diseases* (*ICD-9* and *-10*) codes Used to Identify Fatal and Nonfatal Opioid Overdose Cases **eTable 2.** Association Between Opioid Dose Variability as a Continuous Variable and Opioid Overdose **eTable 3.** Sensitivity Analyses for Nondifferential and Differential Outcome Misclassification Regarding Opioid Dose Variability

## References

[1] Bohnert AS, Logan JE, Ganoczy D, Dowell D A detailed exploration into the association of prescribed opioid dosage and overdose deaths among patients with chronic pain. Med Care. 2016;54(5):-. doi:10.1097/MLR.0000000000000505 26807540 PMC6626611

[2] Bohnert AS, Valenstein M, Bair MJ, Association between opioid prescribing patterns and opioid overdose-related deaths. JAMA. 2011;305(13):1315-1321. doi:10.1001/jama.2011.370 21467284

[3] Dasgupta N, Funk MJ, Proescholdbell S, Hirsch A, Ribisl KM, Marshall S Cohort study of the impact of high-dose opioid analgesics on overdose mortality. Pain Med. 2016;17(1):85-98. 26333030 10.1111/pme.12907

[4] Dunn KM, Saunders KW, Rutter CM, Opioid prescriptions for chronic pain and overdose: a cohort study. Ann Intern Med. 2010;152(2):85-92. doi:10.7326/0003-4819-152-2-201001190-00006 20083827 PMC3000551

[5] Gomes T, Mamdani MM, Dhalla IA, Paterson JM, Juurlink DN Opioid dose and drug-related mortality in patients with nonmalignant pain. Arch Intern Med. 2011;171(7):686-691. doi:10.1001/archinternmed.2011.117 21482846

[6] Dowell D, Haegerich TM, Chou R CDC guideline for prescribing opioids for chronic pain—United States, 2016. MMWR Recomm Rep. 2016;65(1):1-49. 26987082 10.15585/mmwr.rr6501e1

[7] US Department of Veterans Affairs/US Department of Defense VA/DoD Clinical Practice Guideline for Opioid Therapy for Chronic Pain. Washington, DC: US Dept of Veterans Affairs/US Dept of Defense; 2017.

[8] Rosenberg JM, Bilka BM, Wilson SM, Spevak C Opioid therapy for chronic pain: overview of the 2017 US Department of Veterans Affairs and US Department of Defense clinical practice guideline. Pain Med. 2018;19(5):928-941. doi:10.1093/pm/pnx203 29025128

[9] Bohnert ASB, Guy GP Jr, Losby JL Opioid prescribing in the United States before and after the Centers for Disease Control and Prevention’s 2016 opioid guideline. Ann Intern Med. 2018;169(6):367-375. doi:10.7326/M18-1243 30167651 PMC6176709

[10] Guy GP Jr, Zhang K, Bohm MK, Vital Signs: changes in opioid prescribing in the United States, 2006-2015. MMWR Morb Mortal Wkly Rep. 2017;66(26):697-704. doi:10.15585/mmwr.mm6626a4 28683056 PMC5726238

[11] Centers for Disease Control and Prevention *2018 Annual Surveillance Report of Drug-Related Risks and Outcomes—United States: Surveillance Special Report* Washington, DC: Centers for Disease Control and Prevention, US Dept of Health and Human Services; August 31, 2018.

[12] Hedegaard H, Miniño AM, Warner M *Drug Overdose Deaths in the United States, 1999–2017* Hyattsville, MD: National Center for Health Statistics; 2018.

[13] Miller M, Barber CW, Leatherman S, Prescription opioid duration of action and the risk of unintentional overdose among patients receiving opioid therapy. JAMA Intern Med. 2015;175(4):608-615. doi:10.1001/jamainternmed.2014.8071 25686208

[14] Cornish R, Macleod J, Strang J, Vickerman P, Hickman M Risk of death during and after opiate substitution treatment in primary care: prospective observational study in UK General Practice Research Database. BMJ. 2010;341:c5475. doi:10.1136/bmj.c5475 20978062 PMC2965139

[15] Degenhardt L, Randall D, Hall W, Law M, Butler T, Burns L Mortality among clients of a state-wide opioid pharmacotherapy program over 20 years: risk factors and lives saved. Drug Alcohol Depend. 2009;105(1-2):9-15. doi:10.1016/j.drugalcdep.2009.05.021 19608355

[16] Ma J, Bao Y-P, Wang R-J, Effects of medication-assisted treatment on mortality among opioids users: a systematic review and meta-analysis [published online June 22, 2018]. Mol Psychiatry. 29934549 10.1038/s41380-018-0094-5

[17] Sordo L, Barrio G, Bravo MJ, Mortality risk during and after opioid substitution treatment: systematic review and meta-analysis of cohort studies. BMJ. 2017;357:j1550. doi:10.1136/bmj.j1550 28446428 PMC5421454

[18] Binswanger IA, Blatchford PJ, Mueller SR, Stern MF Mortality after prison release: opioid overdose and other causes of death, risk factors, and time trends from 1999 to 2009. Ann Intern Med. 2013;159(9):592-600. doi:10.7326/0003-4819-159-9-201311050-00005 24189594 PMC5242316

[19] Strang J, McCambridge J, Best D, Loss of tolerance and overdose mortality after inpatient opiate detoxification: follow up study. BMJ. 2003;326(7396):959-960. doi:10.1136/bmj.326.7396.959 12727768 PMC153851

[20] Merrall ELC, Bird SM, Hutchinson SJ A record-linkage study of drug-related death and suicide after hospital discharge among drug-treatment clients in Scotland, 1996-2006. Addiction. 2013;108(2):377-384. doi:10.1111/j.1360-0443.2012.04066.x 22925008

[21] Kleiger RE, Stein PK, Bigger JT Jr Heart rate variability: measurement and clinical utility. Ann Noninvasive Electrocardiol. 2005;10(1):88-101. doi:10.1111/j.1542-474X.2005.10101.x 15649244 PMC6932537

[22] Stein PK, Domitrovich PP, Huikuri HV, Kleiger RE; Cast Investigators Traditional and nonlinear heart rate variability are each independently associated with mortality after myocardial infarction. J Cardiovasc Electrophysiol. 2005;16(1):13-20. doi:10.1046/j.1540-8167.2005.04358.x 15673380

[23] Green CA, Perrin NA, Janoff SL, Campbell CI, Chilcoat HD, Coplan PM Assessing the accuracy of opioid overdose and poisoning codes in diagnostic information from electronic health records, claims data, and death records. Pharmacoepidemiol Drug Saf. 2017;26(5):509-517. doi:10.1002/pds.4157 28074520

[24] Langholz B, Clayton D Sampling strategies in nested case-control studies. Environ Health Perspect. 1994;102(suppl 8):47-51. doi:10.1289/ehp.94102s847 7851330 PMC1566552

[25] Langholz B, Goldstein L Risk set sampling in epidemiologic cohort studies. Stat Sci. 1996;11(1):35-53. doi:10.1214/ss/1032209663

[26] Campbell CI, Bahorik AL, VanVeldhuisen P, Weisner C, Rubinstein AL, Ray GT Use of a prescription opioid registry to examine opioid misuse and overdose in an integrated health system. Prev Med. 2018;110:31-37. doi:10.1016/j.ypmed.2018.01.019 29410132 PMC6034705

[27] Glanz JM, Narwaney KJ, Mueller SR, Prediction model for two-year risk of opioid overdose among patients prescribed chronic opioid therapy. J Gen Intern Med. 2018;33(10):1646-1653. doi:10.1007/s11606-017-4288-3 29380216 PMC6153224

[28] Liang Y, Goros MW, Turner BJ Drug overdose: differing risk models for women and men among opioid users with non-cancer pain. Pain Med. 2016;17(12):2268-2279. doi:10.1093/pm/pnw071 28025361 PMC6280954

[29] Zedler B, Xie L, Wang L, Development of a risk index for serious prescription opioid-induced respiratory depression or overdose in Veterans’ Health Administration patients. Pain Med. 2015;16(8):1566-1579. doi:10.1111/pme.12777 26077738 PMC4744747

[30] Deyo RA, Cherkin DC, Ciol MA Adapting a clinical comorbidity index for use with *ICD-9-CM* administrative databases. J Clin Epidemiol. 1992;45(6):613-619. doi:10.1016/0895-4356(92)90133-8 1607900

[31] Quan H, Sundararajan V, Halfon P, Coding algorithms for defining comorbidities in *ICD-9-CM* and *ICD-10* administrative data. Med Care. 2005;43(11):1130-1139. doi:10.1097/01.mlr.0000182534.19832.83 16224307

[32] Hosmer DW Jr, Lemeshow S, Sturdivant RX Applied Logistic Regression. Vol 398 New York, NY: John Wiley & Sons; 2013. doi:10.1002/9781118548387

[33] Cole SR, Chu H, Greenland S Multiple-imputation for measurement-error correction. Int J Epidemiol. 2006;35(4):1074-1081. doi:10.1093/ije/dyl097 16709616

[34] Lyles RH, Tang L, Superak HM, Validation data-based adjustments for outcome misclassification in logistic regression: an illustration. Epidemiology. 2011;22(4):589-597. doi:10.1097/EDE.0b013e3182117c85 21487295 PMC3454464

[35] Rubin DB Multiple Imputation for Nonresponse in Surveys. Vol 81 New York, NY: John Wiley & Sons; 2004.

[36] O’Brien RM A caution regarding rules of thumb for variance inflation factors. Qual Quant. 2007;41(5):673-690. doi:10.1007/s11135-006-9018-6

[37] Frank JW, Levy C, Matlock DD, Patients’ perspectives on tapering of chronic opioid therapy: a qualitative study. Pain Med. 2016;17(10):1838-1847. doi:10.1093/pm/pnw078 27207301 PMC6281041

[38] Kennedy LC, Binswanger IA, Mueller SR, “Those conversations in my experience don’t go well”: a qualitative study of primary care provider experiences tapering long-term opioid medications. Pain Med. 2018;19(11):2201-2211. doi:10.1093/pm/pnx276 29126138 PMC6454789

[39] Berna C, Kulich RJ, Rathmell JP Tapering long-term opioid therapy in chronic noncancer pain: evidence and recommendations for everyday practice. Mayo Clin Proc. 2015;90(6):828-842. doi:10.1016/j.mayocp.2015.04.003 26046416

[40] Warner-Smith M, Darke S, Lynskey M, Hall W Heroin overdose: causes and consequences. Addiction. 2001;96(8):1113-1125. doi:10.1046/j.1360-0443.2001.96811135.x 11487418

[41] White JM, Irvine RJ Mechanisms of fatal opioid overdose. Addiction. 1999;94(7):961-972. doi:10.1046/j.1360-0443.1999.9479612.x 10707430

[42] Young JC, Lund JL, Dasgupta N, Jonsson Funk M Opioid tolerance and clinically recognized opioid poisoning among patients prescribed extended-release long-acting opioids. Pharmacoepidemiol Drug Saf. 2019;28(1):39-47. doi:10.1002/pds.4572 29888409

[43] Cochran G, Gordon AJ, Lo-Ciganic WH, An examination of claims-based predictors of overdose from a large Medicaid program. Med Care. 2017;55(3):291-298. doi:10.1097/MLR.0000000000000676 27984346 PMC5309160

[44] Frank JW, Lovejoy TI, Becker WC, Patient outcomes in dose reduction or discontinuation of long-term opioid therapy: a systematic review. Ann Intern Med. 2017;167(3):181-191. doi:10.7326/M17-0598 28715848

