# Supplementary material for: Association Between Opioid Dose Variability and Opioid Overdose Among Adults Prescribed Long-term Opioid Therapy
Source: JAMA Netw Open. 2019 Apr 19;2(4):e192613. doi: 10.1001/jamanetworkopen.2019.2613 (PMC6481879; doi:10.1001/jamanetworkopen.2019.2613)
Supplement: Supplement. — eTable 1. International Classification of Diseases (ICD-9 and -10) codes Used to Identify Fatal and Nonfatal Opioid Overdose Cases eTable 2. Association Between Opioid Dose Variability as a Continuous Variable and Opioid Overdose eTable 3. Sensitivity Analyses for Nondifferential and Differential Outcome Misclassification Regarding Opioid Dose Variability [file jamanetwopen-2-e192613-s001.pdf]

Supplementary Online Content

Glanz JM, Binswanger IA, Shetterly SM, Narwaney KJ, Xu S. Association between opioid dose variability and opioid overdose among adults prescribed long-term opioid therapy. *JAMA Netw Open*. 2019;2(4):e192613. doi:10.1001/jamanetworkopen.2019.2613

eTable 1. *International Classification of Diseases* (*ICD-9* and *-10*) codes Used to Identify Fatal and Nonfatal Opioid Overdose Cases

eTable 2. Association Between Opioid Dose Variability as a Continuous Variable and Opioid Overdose

eTable 3. Sensitivity Analyses for Nondifferential and Differential Outcome Misclassification Regarding Opioid Dose Variability

This supplementary material has been provided by the authors to give readers additional information about their work.

© 2019 Glanz JM et al. *JAMA Network Open.*

eTable 1. *International Classification of Diseases* (*ICD-9* and *-10*) codes Used to Identify Fatal and Nonfatal Opioid Overdose Cases

**Overdose Outcome**	**ICD-9 codes**	**ICD-10 codes**
Non-fatal, due to heroin	965.01, E850.0	T40.1X1, T40.1X2, T40.1X3, T40.1X4
Fatal, due to heroin		Underlying cause of death codes for drug poisoning (X40–X44, X60–X64, X85, Y10–Y14), and a contributing cause of death code indicating heroin involvement (T40.1)
Non-fatal, due to pharmaceutical opioids	965.0, 965.00, 965.02, 965.09, E850.1, E850.2	T40.0X1, T40.0X2, T40.0X3, T40.0X4, T40.2X1, T40.2X2, T40.2X3, T40.2X4, T40.3X1, T40.3X2, T40.3X3, T40.3X4, T40.4X1, T40.4X2, T40.4X3, T40.4X4
Fatal, due to pharmaceutical opioids		Underlying cause of death codes for drug poisoning (X40–X44, X60–X64, X85, Y10–Y14), and a contributing cause of death code indicating pharmaceutical opioid involvement (T40.2, T40.3, T40.4)

© 2019 Glanz JM et al. *JAMA Network Open.*

eTable 2. Association Between Opioid Dose Variability as a Continuous Variable and Opioid Overdose

**Characteristic**	**Adjusted mOR (95% CIs)[a]**
Variability in dose (SD)	1.00 (1.00, 1.01)[c]
Dose[b]	
0-20 MME	1.00
21-50 MME	1.49 (0.95, 2.35)
51-100 MME	2.50 (1.53, 4.08)
>100 MME	2.83 (1.69, 4.74)
Age, y	
≤45	1.00
46-55	0.61 (0.40, 0.92)
56-65	0.82 (0.55, 1.24)
>65	0.72 (0.45, 1.16)
Sex	
Female	1.00
Male	0.85 (0.62, 1.15)
Hispanic ethnicity	
Yes	1.00
No	0.90 (0.56, 1.43)
Race	
White	1.00
Black	1.36 (0.73, 2.52)
Other	1.31 (0.77, 2.24)
Drug or alcohol use disorder	
No	1.00
Yes	2.29 (1.67, 3.16)

© 2019 Glanz JM et al. *JAMA Network Open.*

Tobacco use or use disorder	
No	1.00
Yes	1.69 (1.20, 2.38)
Former	1.07 (0.72, 1.58)
Mental health disorder diagnosis	
No	1.00
Yes	3.02 (1.59, 5.74)
Opioid formulation	
Short-acting	1.00
Long-acting/extended-release/Both	1.29 (0.87, 1.93)
Benzodiazepine dispensing	
No	1.00
Yes	1.96 (1.44, 2.66)
Modified Charlson Comorbidity Index	1.26 (1.17, 1.36)

Abbreviation: MME, milligrams morphine equivalent; mOR, matched odds ratio; CI, confidence interval; SD, standard deviation

[a]Matched on index dose, calendar time, and length of follow-up

[b]In the 3 months before the index date (date of overdose from cases and matched date for controls)

[c]p-value=0.018

© 2019 Glanz JM et al. *JAMA Network Open.*

eTable 3. Sensitivity Analyses for Nondifferential and Differential Misclassification Regarding Opioid Dose Variability

Characteristic	Adjusted mORs (95% CIs)	Adjusted mORs (95% CIs) with 19% nondifferential misclassification rate	Adjusted mORs (95% CIs) with 19% differential misclassification rate[a]
Variability in dose (SD)			
0-5.3	1.00	1.00	1.00
5.4-9.1	1.43 (0.68, 3.01)	1.44 (0.62, 3.31)	1.41(0.67, 2.98)
9.2-14.6	1.61 (0.79, 3.31)	1.61 (0.72, 3.61)	1.58 (0.76, 3.27)
14.7-27.2	2.19 (1.08, 4.43)	2.15 (0.97, 4.76)	1.57 (0.75, 3.29)
>27.2	3.32 (1.63, 6.77)	3.23 (1.45, 7.23)	2.32 (1.10, 4.90)
Dose in 3 months before index date			
0-20 MME	1.00	1.00	1.00
21-50 MME	1.53 (0.97, 2.40)	1.53 (0.92, 2.53)	1.53 (0.94, 2.50)
51-100 MME	2.27 (1.39, 3.70)	2.30 (1.34. 3.97)	2.25 (1.31, 3.87)
>100 MME	2.37 (1.41, 3.98)	2.42 (1.35, 4.32)	2.49 (1.38, 4.49)
Age			
≤45	1.00	1.00	1.00
46-55	0.64 (0.42, 0.96)	0.64 (0.41, 1.01)	0.63 (0.40, 0.98)
56-65	0.83 (0.55, 1.25)	0.83 (0.52, 1.32)	0.78 (0.49, 1.23)
>65	0.73 (0.45, 1.18)	0.71 (0.42, 1.22)	0.69 (0.41, 1.17)
Gender			
Female	1.00	1.00	1.00
Male	0.87 (0.64, 1.18)	0.87 (0.62, 1.23)	0.86 (0.61, 1.21)
Hispanic ethnicity			
Yes	1.00	1.00	1.00
No	0.88 (0.55, 1.41)	0.88 (0.52, 1.49)	0.87 (0.52, 1.46)

© 2019 Glanz JM et al. *JAMA Network Open.*

Race			
White	1.00	1.00	1.00
Black	1.43 (0.77, 2.65)	1.40 (0.70, 2.79)	1.29 (0.64, 2.58)
Other	1.37 (0.80, 2.36)	1.21 (0.65, 2.24)	1.17 (0.63, 2.15)
Drug or alcohol use disorder	2.18 (1.58, 3.00)	2.18 (1.52, 3.11)	2.20 (1.55, 3.14)
Tobacco use or use disorder			
No	1.00	1.00	1.00
Yes	1.68 (1.19, 2.37)	1.70 (1.16, 2.50)	1.64 (1.12, 2.41)
Former	1.05 (0.71, 1.56)	1.09 (0.70, 1.69)	1.08 (0.70, 1.66)
Mental health disorder diagnosis			
No	1.00	1.00	1.00
Yes	2.97 (1.57, 5.64)	3.12 (1.51, 6.45)	3.19 (1.55, 6.55)
Opioid formulation			
Short-acting	1.00	1.00	1.00
Long acting/extended-release/both	1.23 (0.83, 1.84)	1.24 (0.80, 1.93)	1.30 (0.84, 2.03)
Benzodiazepine dispensing			
No	1.00	1.00	1.00
Yes	1.90 (1.40, 2.59)	1.91 (1.36, 2.70)	1.89 (1.35, 2.66)
Modified Charlson Comorbidity Index	1.25 (1.16, 1.35)	1.26 (1.16, 1.36)	1.25 (1.15, 1.36)

[a]There are 168 cases in the two highest variability groups and 60 in the rest of the variability groups. We assumed 25.9% misclassification rate in the two highest variability groups and 0% misclassification rate for the cases in the rest of the variability groups while maintaining an overall 19% misclassification rate.

© 2019 Glanz JM et al. *JAMA Network Open.*
